# Use of T-Scan III in analyzing occlusal changes in molar fixed denture placement

**DOI:** 10.1186/s12903-024-04014-1

**Published:** 2024-02-22

**Authors:** Hei Chan, Adili Alimujiang, Sin Iok Fong, Ming-Le Wu, Ran Liang, Peng-Yu Lai, Hui-Wen Wei, Shan Shen

**Affiliations:** 1 School of Stomatology, Jinan University, Guangzhou City, 510632 Guangdong Province China; 2 Department of Stomatology, Affiliated Stomatological Hospital of Jinan University (Daliang Hospital Foshan City), Shunde District, Foshan City, 528399 Guangdong Province China; 3 Department of Stomatology, The First Affiliated Hospital of Jinan University, No. 613, Huangpu Avenue West, Tianhe District, Guangzhou, 510632 China

**Keywords:** Fixed denture, Occlusion adjustment, Occlusal force, T-SCAN

## Abstract

**Background:**

This study aims to analyze the longitudinal variation of occlusal force distribution prior to and after fixed restoration for molar full-crowns with T-SCAN III which provide reference for occlusal adjustment and long-term maintenance.

**Methods:**

We enrolled a total of 20 patients who received conventional restorative treatment for molars. The occlusion examination was conducted in 3 stages (before placement, immediately after placement, and 3 months after placement) using T-SCAN III (Tekscan South Boston, MA, USA, 10.0) to examine and measure the occlusal contact areas of the full dentition.

**Results:**

The results indicated that the occlusal force distribution in the molar region of the patients changed before and after the fixed restoration, but the percentages of occlusal force in the dental arch of the molar did not differ significantly before and after the restoration (*P* > 0.05). Three months after the fixed restoration, the percentage of occlusal force in the restored dental arches of lateral teeth increased significantly (*P* < 0.05).

**Conclusion:**

The results of this study indicated that the occlusal forces of the patients changed with tooth movement and adaptation, which is mainly reflected in the increasing occlusal force. Quantitative occlusal force analysis using T-SCAN III occlusal analyzer can provide more objective and accurate data to effectively guide clinical occlusion adjustments.

**Supplementary Information:**

The online version contains supplementary material available at 10.1186/s12903-024-04014-1.

## Background

Tooth loss and damage have a direct effect on occlusion. A good occlusal relationship is also essential for the coordination of the physiological functions of the oral-maxillary system. The purpose of dental restoration is to restore the occlusal relationship to its original state. Occlusal analysis is important in the restoration process [1]. If the occlusal premature contact of a restoration exceeds the physiological limits of the patient, not only can the restoration cause tooth sensitivity, but it can also worsen periodontal disease, leading to masticatory muscle pain and temporomandibular disorders (TMDs) [2, 3]. With the advancement of technology and the development of society, increased demands have emerged for the identification and mitigation of occlusal interferences, and the success rate of restorative treatment has increased. It is important to obtain reliable occlusion records and ensure proper occlusal contact in restorative dentistry [4]. Obtaining an accurate occlusion record and communicating it to the dental technician is vital for making prosthetics and restorative treatment [5]. In dental treatment, occlusion adjustment refers to the selection and grinding of occlusal premature contact, removal of premature contact, adjustment of the patient’s occlusal contact and muscle tissues, and coordination of the temporomandibular joint and central nervous system, thereby restoring normal physiological function and oral and maxillofacial systems.

**Fig. 1:**
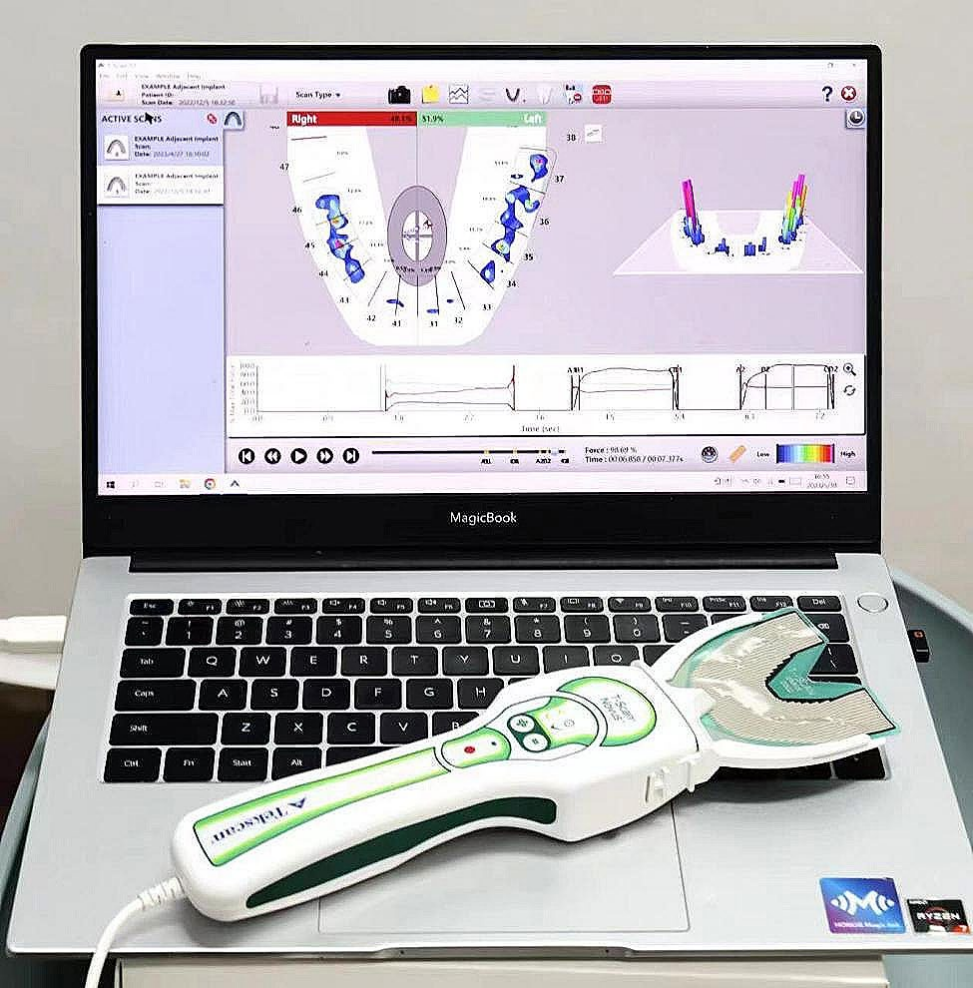
Tekscan novus system

**Fig. 2:**
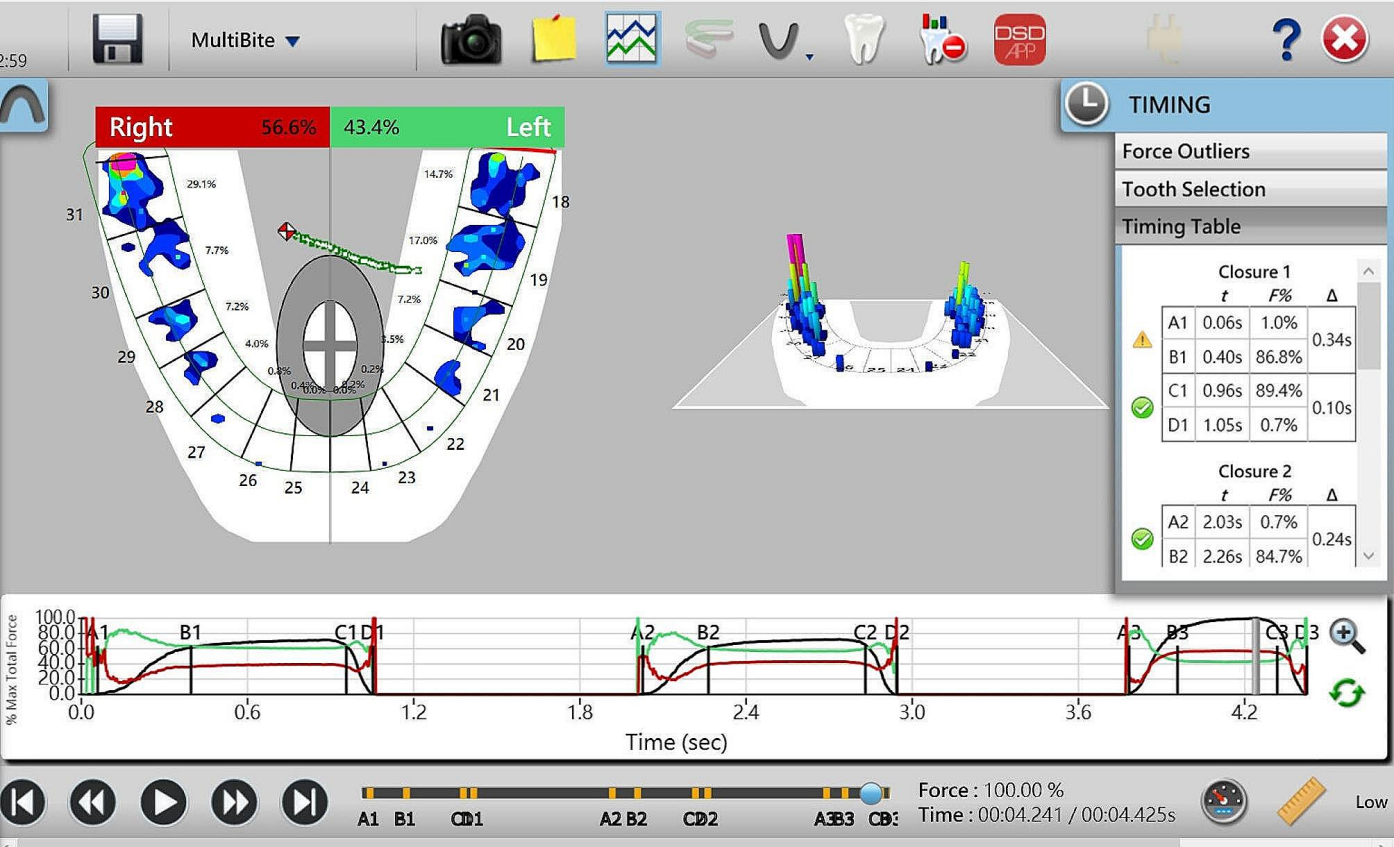
T-Scan 2D & 3D image information &time parameters

With the development of 3D scanning techniques,quantitative and optimal results make it possible to achieve accurate occlusion relationgship [6–8], such asT-SCAN, etc. T-SCAN (Tekscan South Boston, MA, USA, 10.0) is one of the most effective tools for analyzing occlusal contact, occlusal time, and their relationship (Figs. 1 and 2). It detects, in increments of 0.003 s, the sequence of each tooth contact, the position of force on each tooth surface at the point of contact, the percentage of relative occlusal force, and the trajectory center of resultant force. Using T-SCAN III, Kerstein accurately analyzed the stress distribution in the dental arch, the maximum occlusal force, and the initial contact to determine if the occlusal stress contact is destructive [9]. This system uses dynamic images to measure the contact position and excessive stress distribution at the initial contact point. If excessive stress can be precisely eliminated, the lifespan of prostheses can be extended. Also, the occlusal sensing film scanned by T-SCAN, has no impact on occlusal force, is unaffected by saliva and can be reused 15 to 25 times [10, 11]. 

The purpose of this study was to examine the effect of crown and bridge restoration treatment for a single molar on occlusal force redistribution and to assess the changes in occlusal force distribution of the restoration and the possible influencing factors at each stage, as well as to investigate the feasibility of using T-Scan to quantify the occlusal contacts of the restorations and provide more objective and accurate information to assist patients in restoring stable occlusal function.

## Materials and methods

In this prospective study, we conducted occlusal measurements at 3 stages: preoperative, postoperative, and the third month after surgery. This study was approved by the Medical Ethics Committee of Ji’nan University (approval number. JNUKY-2022-045). All patients included in the study signed the surgical informed consent form.

### Sample size calculation

The sample size was determined using experimental research methodology [12]. The standard deviation σ was 2.5 units, the two sides α was 0.05, and the test efficiency of 1-β was 0.90 for the difference in occlusal force of the patients in this study. According to the calculations, with a standard deviation of 2.5 units for the sample occlusal force (s = σ = 2.5), a standard deviation of 2 units for the change in occlusal force (δ = 2), an α level of 0.05, a Zα/2 value of 1.96, a β level of 0.10, and a Zβ value of 1.282, the required sample size was 16.42, meaning that at least 17 participants were needed for the study. The sample size was calculated according to the formula:

$$n ={\left[\frac{\left({\text{Z}}_{{\upalpha }/2}+{\text{Z}}_{{\upbeta }}\right)\text{S}}{{\updelta }}\right]}^{2}$$

The asymmetry occlusal force index (AOF) reveals the difference in occlusal force between the left and right sides and is calculated as follows [13]:

AOF (%) = occlusal force of left side - occlusal force of right side/total occlusal force × 100%.

### Inclusion and exclusion criteria

Patients older than 18 years with good periodontal health and no more than 3 units of single crown or fixed denture restorations in the molar region met the inclusion criteria.

Patients were excluded from the study if they presented with TMJ pain, had a history of periodontitis, used analgesics and anesthetics during the observation period, received treatment including occlusion adjustment, orthodontic treatment, extraction or implant restoration.

### Occlusal parameters

**Fig. 3:**
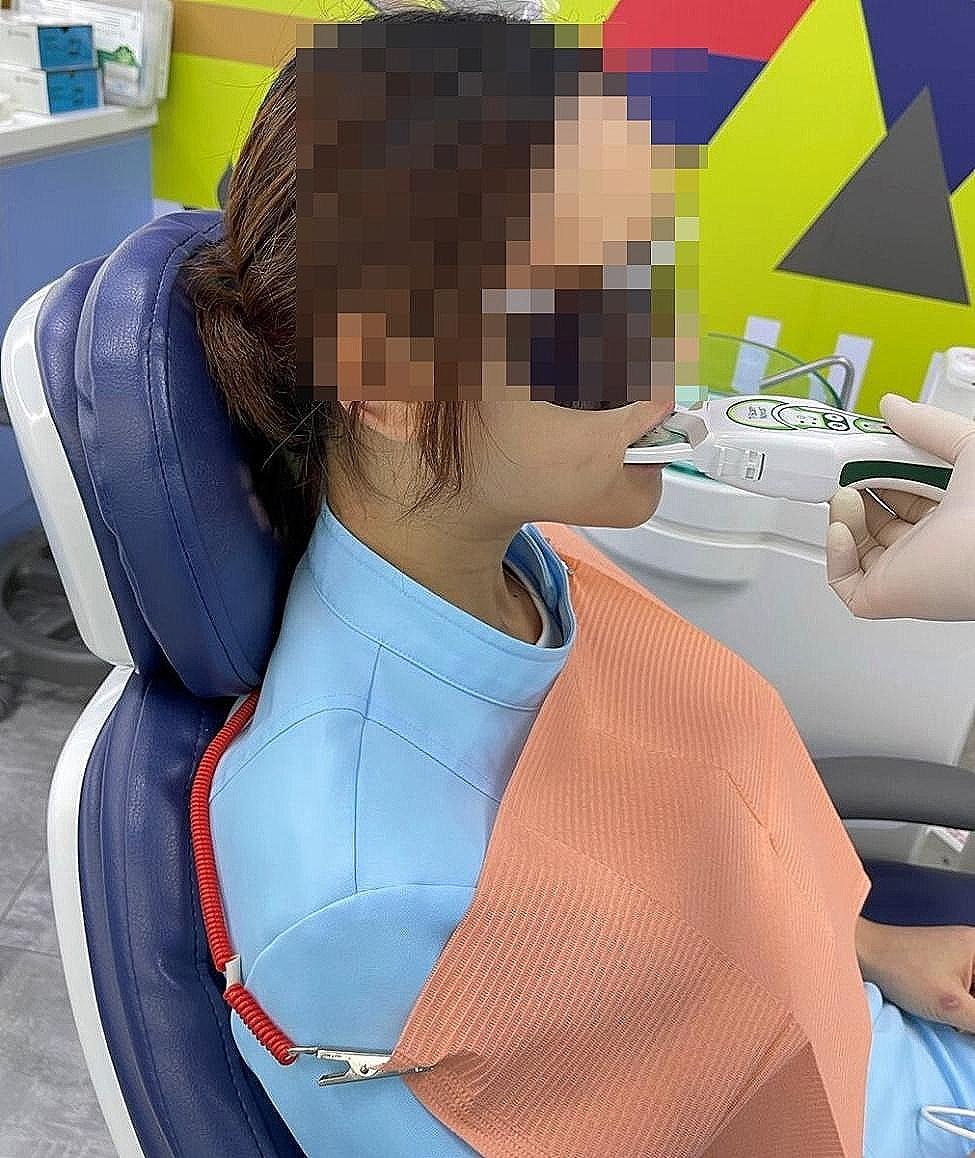
Patient with chair in sitting position

**Fig. 4:**
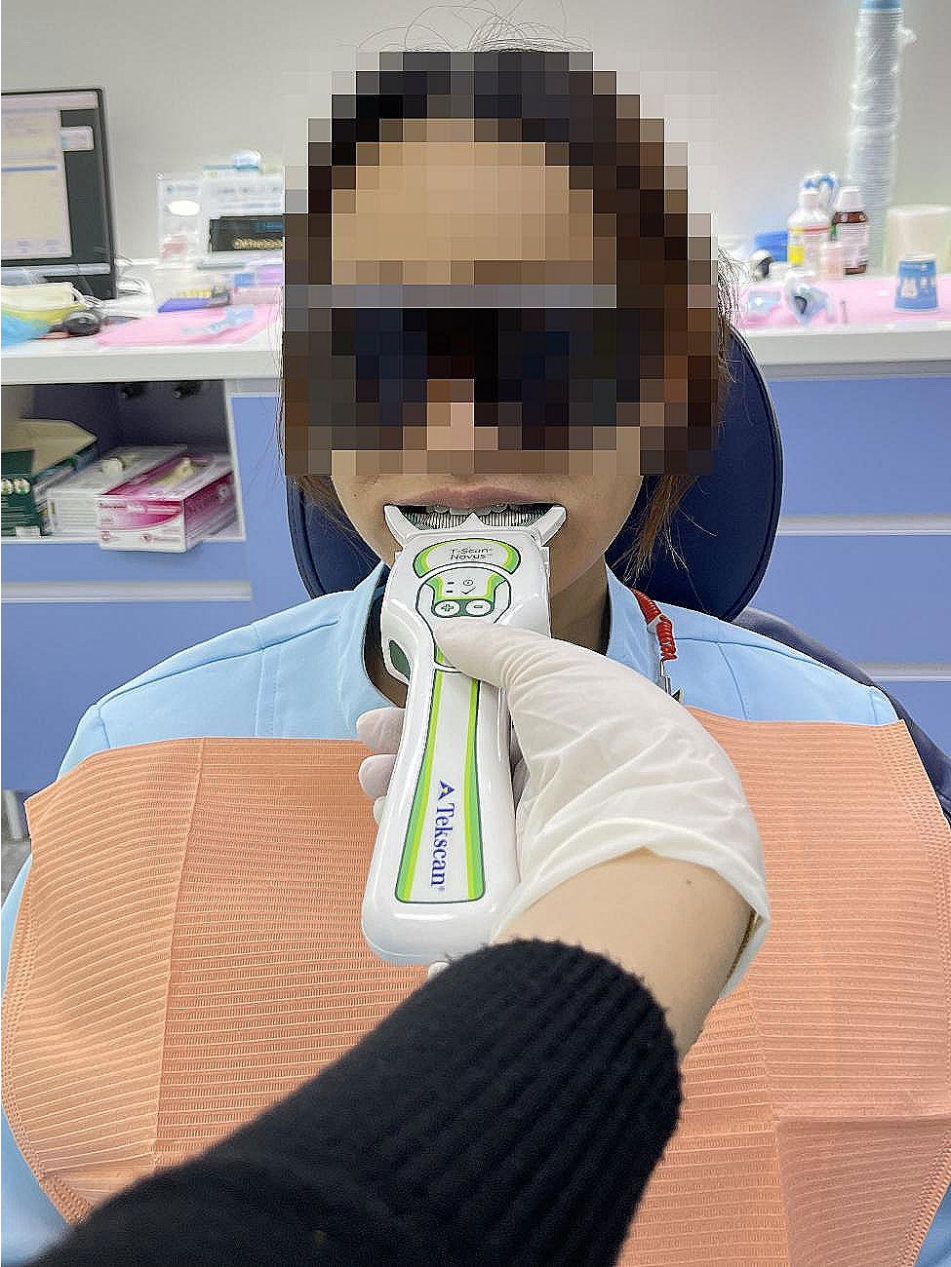
Place T-scan handle uniform bite in ICP 3 times was recorded

On the day of the visit, prior to crown placement, the patients were seated upright in a dental chair (with the Frankfort horizontal plane parallel to the floor) (Fig. 3). The T-SCAN III (Tekscan South Boston, MA, USA, 10.0) was then inserted into the mouth of the patients, and they were instructed to bite firmly three times at the maximum cusp intersection position (MIP) (Fig. 4).

Dental preparation was performed according to the fixed restoration benchmarks (1.5–2.0 mm occlusal restoration, 1.0–1.5 mm axial restoration, and marginal position no more than 0.5 mm subgingival). Impressions were made, and crown and bridge restorations were made. Then, the dental surgeon used articulating paper (Arti-Check BK09, Bausch, USA) for occlusion adjustment.

At 30 min post-crown placement and at the 3-month postoperative follow-up, the digital sensor (T-SCAN III) was reused, and the average of the 3 measurements was calculated for analysis. The patients’ responses to the standard questionnaire used in the study (Questionnaire 1), formulated based on the questionnaire developed by Chaithanya [14], were also recorded. The questionnaire was designed to measure (1) how well the teeth fit together, (2) the level of occlusal discomfort, and (3) chewing efficiency during treatment.

### Statistical analysis

Statistical analyses were performed using SPSS 26.0 software with continuous type variables that follow normal distribution expressed as mean ± standard deviation. Percentage of occlusal force, Occlusion time, and Disclusion time of the mesial and distal adjecent teeth of the restored arch, contralateral arch, and restored tooth position were tested using paired *t*-tests with a test level of α = 0.05 (bilaterally) for the restored arch, contralateral arch, restored tooth position, and for the mesial and distal adjecent teeth of the restored arch, pre-restorations, and post-restorations. For samples that did not follow the normal distribution, the data were expressed as median (25th, 75th percentile), and differences between the groups were compared using Wilcoxon signed rank-sum test.

## Results

We enrolled 20 patients (11 females and 9 males, all over 18 years of age with a mean age of 44.55 ± 12.13 years) who received crown or bridge restorative treatment for molars at the Zenith Dental Clinic in Macau between June 2020 and December 2021. Details of gender, age, position of the restored teeth, and history of smoking and alcohol consumption are provided in Table 1.

**Table 1:** Patient information and restoration tooth position

Gender	Age	Tooth position	Smoke	Quantity	Drinks
F	59	46	No	No	No
F	37	45,46,47	No	No	No
M	41	47	No	No	No
F	33	26	No	No	No
F	33	15	No	No	No
M	65	17	No	No	Occasionally
M	34	37	Yes	10/day	Occasionally
M	49	47	Yes	5/day	Occasionally
M	63	36	Yes	20/day	Occasionally
F	28	25	No	No	Occasionally
M	54	27	No	No	No
F	38	36	No	No	No
F	68	26	No	No	No
F	31	44	No	No	No
F	43	16	No	No	No
F	35	25	No	No	No
M	38	27	No	No	No
M	36	36	Yes	5/day	Occasionally
M	56	26	No	No	No
F	50	46	No	No	No

Due to the COVID-19 epidemic delaying the follow-up time and increasing the missed visit rate, 15 of the 20 patients (8 females, 7 males, mean age of 46.33 ± 13.56 years) successfully completed the 3-month follow-up, and the data were statistically reanalyzed.

### Comparison of occlusal contact before and after fixed restorations

Table 2 displays the T-SCAN occlusal data of patients before and after fixed restorations. The percentage of occlusal force on both sides of the arch did not change significantly after restoration (*P* > 0.05). However, the percentage of occlusal force of mesial adjacent teeth increased slightly after restoration [(7.94 ± 1.82)%], as compared to before restoration [(6.27 ± 1.44)%], with no statistically significant difference observed (*P* > 0.05). The percentage of occlusal force before and after restoration of restored tooth position was (10.15 ± 2.33)% and (9.08 ± 2.08)%, respectively, with no significant difference (*P* > 0.05). The percentage of occlusal force of the distal adjacent teeth was (11.98 ± 2.75)% before restoration, and (13.10 ± 3.01)% after restoration, with a slight increase after restoration, but the difference was not statistically significant.

**Table 2:** Occlusion changes in indicators before and after crown restoration ($\bar x \pm s$)

Part	Before (*N* = 20)	After (*N* = 20)	t	*P*
Arch with fixed restoration, %	46.15 ± 17.40	46.75 ± 15.78	-0.214	0.833
Arch without fixed restoration, %	53.86 ± 17.40	53.25 ± 15.78	0.214	0.833
Fixed denture, %	10.15 ± 2.33	9.08 ± 2.08	0.053	0.959
Mesial teeth, %	6.27 ± 1.44	7.94 ± 1.82	-1.262	0.223
Distal teeth, %	11.98 ± 2.75	13.10 ± 3.01	-0.337	0.740

### Effect of articulating paper on the occlusal force of the restorations

**Fig. 5:**
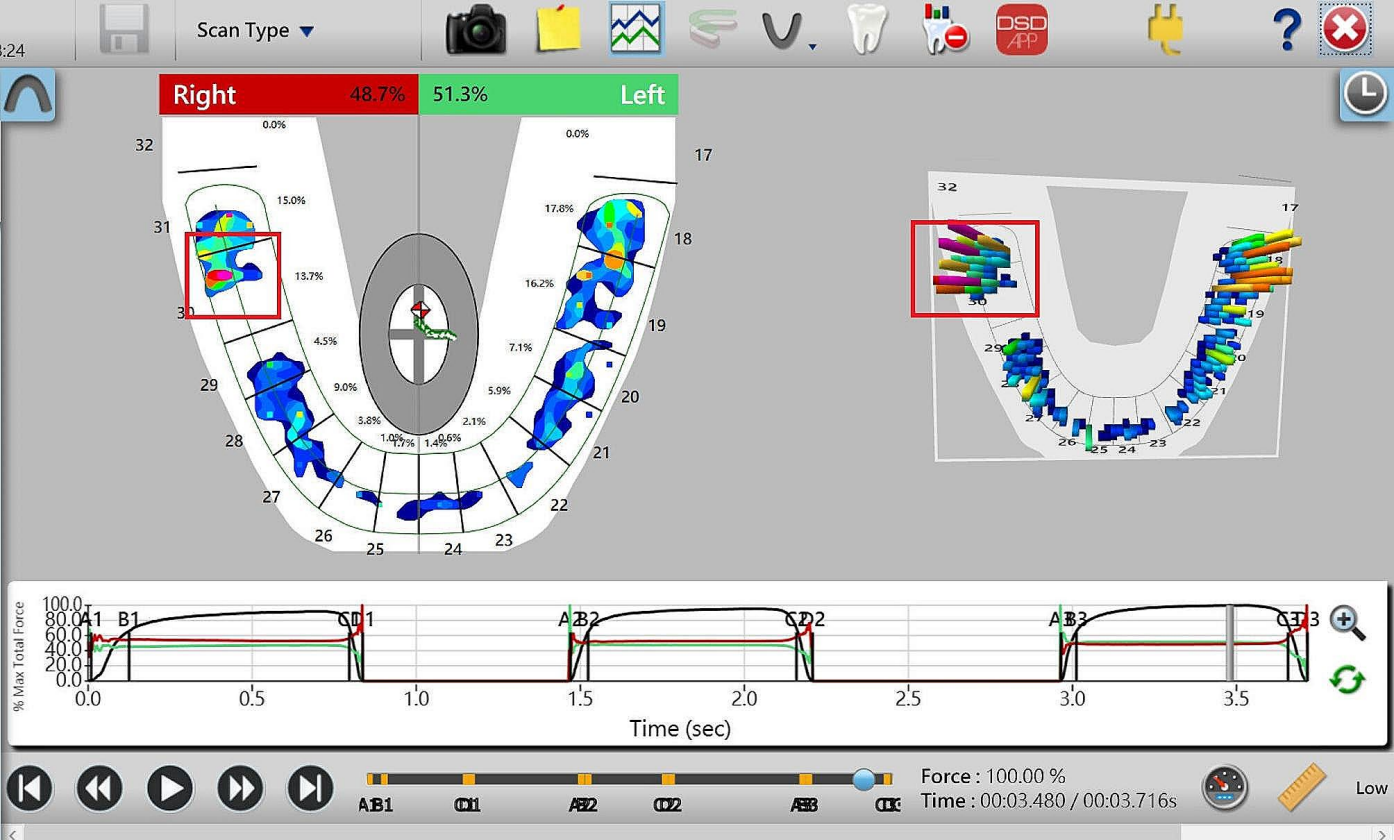
After crown cementation and adjustment, T-scan III 2D and 3D information

Although restoration was performed under articulating paper guidance until both the patient and the dentist were satisfied, 15% of patients still had occlusal discordance, as shown in Fig. 5.

### Analysis of occlusal contact by the follow-up at the third month after surgery

The T-SCAN occlusal information after restoration is presented in Table 3. The percentage of occlusal force of restored arch 3 months after restoration was (51.99 ± 11.20)%, which was significantly higher than the (45.75 ± 16.14)% before restoration, (*P* < 0.05). The percentage of occlusal force in the contralateral arch was (56.04 ± 12.86)% before restoration, and (48.08 ± 11.19)% 3 months after restoration (*P* < 0.05). The percentage of occlusal force of restored teeth at 3 months after restoration was (12.02 ± 9.22) %, which was higher than that before restoration (9.89 ± 8.44) %, but with no significant difference (*P* > 0.05).

**Table 3:** Occlusion changes in indicators before and after 3 months of crown restoration

Part	Before (*N* = 15)	After (*N* = 15)	t/Z	*P*
Arch with fixed restoration, %	45.75 ± 16.14	51.99 ± 11.20	-2.652	0.019*
Arch without fixed restoration, %	56.04 ± 12.86	48.08 ± 11.19	3.058	0.009**
Fixed denture, %	9.89 ± 8.44	12.02 ± 9.22	-1.488	0.159
Mesial teeth %	4.50 (2.70,9.70)	7.00 (4.70,12.10)	-2.528	0.011*
Distal teeth %	9.10 (0.00,20.20)	8.90 (0.00,24.80)	-1.601	0.109

The percentage of occlusal force [7.00 (4.70, 12.10)] of the mesial adjacent teeth increased significantly (*P* < 0.05) 3 months after restoration compared to that [4.50 (2.70, 9.70)] before restoration; the percentage of occlusal force [8.90 (0.00,24.80)] of the distal adjacent teeth increased after restoration, but the difference was not statistically significant (*P* > 0.05).

### Data analysis of occlusion time and disclusion time

The Occlusion Time before and after bonding was (0.37 ± 0.08) s and (0.23 ± 0.05) s, respectively, and the Occlusion Time was significantly shorter after bonding, but the difference was not statistically significant (*P* > 0.05); the Occlusion Time 3 months after restoration was (0.28 ± 0.20) s.

The mean Occlusion Time was (0.17 ± 0.04) s before and (0.21 ± 0.05) s after restoration, with increased disclusion time, but the difference was not significantly different (*P* > 0.05). There was no significant difference between the time to prosthesis detachment in the immediate postoperative period and 3 months post-surgery.

Based on the Occlusion time results (Table 4), the coordination of the muscle position with the maximum cusp interposition improved after the restoration. Also, the occlusal contact of the restored tooth improved over time, approaching the manufacturer-recommended occlusion time standard after 3 months. Thus, the fixed restoration assisted in restoring the occlusal force and coordination of masticatory muscles.

**Table 4:** OT, DT at three time points

	Before	Immediately	Three months later	F	*P*
OT (s)	0.48 s ± 0.40 s	0.38 s ± 0.23 s	0.28 s ± 0.20 s	1.750	0.195
DT (s)	0.23 s ± 0.18 s	0.21 s ± 0.25 s	0.17 s ± 0.08 s	0.537	0.591

### Relationship between fixed prosthetic occlusion and comfort

Subjective analysis 3 months after the restoration revealed that the occlusion comfort and masticatory efficiency improved significantly, the eating habits of the patients improved, with 80% of patients being able to chew hard foods, and their sensory balance contact at the back of the restored teeth increased from 26 to 80% before and after the restoration. Patients were pleased with the therapeutic outcomes, which achieved the therapeutic objectives (Table 5).

**Table 5:** Comparison of patient’s response before versus after prosthetic treatment

Question	Situation	Before prosthetic treatment	After prosthetic treatment
Q1 Comfort on biting on teeth lightly	I feel comfortable	66.6%	93.3%
Q2 Comfort while biting hard on back teeth in maximum intercuspation	I feel comfortable	33.3%	80%
Q3 Even contact of back teeth when biting hard	Have contact	40%	86.7%
Q4 Comfort while biting on quadrant with fixed denture	I feel comfortable	20%	93.3%
Q5 Even contact of back teeth when biting hard on quadrant with missing teeth	Have contact	26.6%	80%
Q6 Pain while biting down hard on teeth in maxixum intercuspation	No pain	53%	80%
Q7 Pain while biting hard on back teeth on quadrant with missing teeth	No pain	40%	86.7%
Q8 Pain/tenderness during chewing at jaw joint corresponding to quadrant with missing/restored tooth	No pain	46.7%	66.7%
Q9 Ability to chew tough food such as meat with back teeth	Chew very well	26.7%	86.7%
Q10 Ability to chew fresh fruits such as apple with back teeth	Chew very well	20%	66.7%
Q11 Change in eating habits before and after the restoration	Mainly soft foods, such as fish, bananas	80%	26.7%
Q12 Change in eating habits before and after the restoration	Mainly tough foods, such as beef, apples	20%	73.3%

### Comparison of T-SCAN and articulating paper in adjusting the occlusion

The occlusion was adjusted using articulating paper and T-SCAN and both groups were given questionnaires. In terms of efficiency and comfort, the results revealed that T-SCAN-guided occlusion adjustment was superior to articulating paper-guided occlusion adjustment (Table 6).

**Table 6:** Comparison of tekscan and articulating paper occlusal adjustment

Question		Articulating paper	T-SCANIII
1. Clinical time required to adjust occlusion	15 min or less	10	6
	15–30 min	10	14
	More than 30 min	0	0
2. Frequency required to adjust occlusion	1–3 times	1	18
	3–6 times	14	2
	6 times or more	5	0
3. Number of return visits needed to adjust occlusion	1 time	12	18
	2 time	8	2
	3 time	0	0
4. Comfort while biting after adjusting occlusion	Comfortable	10	18
	Have discomfort	6	0
	Not sure	4	2
5. Mastication after adjusting occlusion	Chew very well	12	18
	Cant chew well	8	0
	Not sure	0	2

### Asymmetry occlusal force index

The mean asymmetry occlusal force index was 20.09% prior to restoration, 20.48% after treatment, and 17.11% three months after treatment. The asymmetry occlusal force index was not significantly different at the three time points, but it decreased significantly after three months and approached the mean value of the normal population [14]. This may be due to the short-term lateralized masticatory habits of the patients because of discomfort during treatment and the inability of the occlusal forces on both sides to adapt to this habit after surgery. Three months after restoration, the masticatory muscle groups were appropriately exercised, the occlusal force of the restored side was improved, and the difference in bilateral occlusal force decreased.

## Discussion

In natural dentition, several factors, such as tooth wear, periodontal disease, TMDs, orthodontic treatment, and tooth eruption, can influence occlusal force distribution and occlusal contact [15, 16]. In this study, we excluded factors such as severe periodontal disease, TMDs, and orthodontic treatment that can alter occlusal forces and focused on local occlusal changes of the fixed restorations.

After restorative treatment, the dental defect is repaired, and the occlusal force should increase. The results of this study indicated that 30 min after restoration, the occlusal force of the restoration did not increase, but decreased. This may be because the restored abutment must withstand not only the maximum occlusal force it can withstand, but also the maximum occlusal force of the contralateral molar transferred by the bridge and crown, which exceeds the abutment’s own bearing capacity. The body inhibits the masticatory muscles on the restorative side through a feedback mechanism that exists between the periodontal receptors, the center, and the masticatory muscles, thus reducing the maximum resultant force exerted on the fixed abutment to a level that the abutment can withstand. Simultaneously, the maximum resultant force acting on the remaining teeth on the restorative side is reduced. Also, many patients experience discomfort and limitations on the affected side due to a fractured or missing tooth, limiting their ability to chew on that side prior to treatment. As a result, short-term unilateral mastication occurs. Considering all the above factors, we hypothesize that lateral chewing caused an imbalance in the occlusion of both sides of the dental arch of the molar following full-crown restoration, resulting in a greater occlusal force and contact area on the non-restored side of the arch.

The percentage of occlusal force of the restored teeth increased significantly after three months, due to the adaptability of the lateral side of the restoration to the new occlusal contact. For fixed abutment teeth, the change in occlusal force altered the periodontal support tissues and substantially increased the support capacity of the teeth with an abutment, increasing the maximum occlusal force within their bearing capacity and restoring the maximum occlusal force of the other teeth. Thereby, the percentage of occlusal force increased in the adjacent teeth to the restorations, but the difference was not significant because some of the restorations were located at the free end and the distal adjacent teeth were not present.

Zheng investigated the changes in occlusal forces of full-crown restorations and analyzed the occlusal forces of patients before placement, immediately after placement, and 2 months, 4 months, and 6 months after restoration; the results demonstrated that the occlusal force of both the restoration and the restoration side decreased, which is consistent with our findings [17]. Specific results indicate that the occlusal force on both sides of the arch began to decrease immediately after restoration, and after 4 months, the occlusal force on both sides of the arch returned to the pre-restoration level. Also, 6 months after the restoration, the occlusal force on the restored side increased significantly. The study by Zheng also indicated that, over time, psychological adaptation and dental treatment reduced patients’ anxiety, allowing them to re-adjust to chewing and regain their occlusal force.

Although restoration was performed under articulating paper guidance until both the patient and the dentist were satisfied, T-SCAN revealed that 15% of patients still exhibited occlusal discordance. Articulating paper was used for static occlusal force detection. According to numerous occlusion textbooks, the marked area represents the load contained within the marker [18]. Large, dark markers are recommended for heavy occlusal loads, whereas small, light markers are recommended to indicate light occlusal loads [19]. However, research has revealed a low correlation between articulating paper markers and the T-SCAN occlusal force concentration regions. Frequently, a small marker indicates a high force concentration. However, a large, dark marker does not indicate heavy force.

Articulating paper-oriented orthodontics also depends on the patient’s occlusal sensory feedback, which can be variable. When using articulating paper, patients must repeatedly bite to obtain occlusal marks. However, repeated occlusion fatigues the masticatory muscle and reduces the pressure perception in the periodontal ligament, which may result in deviations [20]. Also, after tooth extraction, important sensory conduction during mastication is lost due to the disappearance of receptors in periodontal ligaments [21]. These physiological sensations prevent the tooth and oral system from sensing unfavorable occlusal forces, which can lead to restoration failure and tooth loss [21, 22]. 

Advancements in dentistry have increased the need for visualization of dental and associated for the diagnosis and treatment [6]. Unlike traditional occlusal examination methods such as articulating paper, T-SCAN can accurately obtain occlusal force distributions, relative occlusal force values, and occlusal time series without relying on the psychological influence of the patient and the physician [23]. The quantitative occlusal images generated can be viewed multiple times by doctors without requiring the patient to bite repeatedly. Thus, T-SCAN III facilitates more precise occlusal alignment. These benefits have made it applicable to a wide range of oral restorations, allowing for a more thorough analysis of the distribution and changes in occlusal force throughout the treatment process.

The T-SCAN system can also measure OcclusionTime and Disclusion Time to determine changes [11, 24, 25]. A comprehensive analysis of occlusal contact characteristics using this system can be performed for clinical and scientific purposes. Occlusion Time was found to be directly related to the patient’s occlusal contact pattern [26] and is believed to accurately reflect occlusion [27], whereas Disclusion Time is related to tooth contact and muscle activity [28]. Abnormal Disclusion Time can result in alterations in muscle activity, which may lead to TMDs [29]. This enables dentists to detect and precisely adjust subtle interferences by patients to improve the occlusion of dental implants.

This study is limited by the potential for bias due to its small sample size and short follow-up period. The rate of missed appointments after 3 months was 25% (5 out of 20 cases), suggesting that this was only a pilot study. As T-SCAN utilizes force percentages rather than absolute force values, and because combined treatment plans and long-term efficacy are influenced by a number of factors, larger sample sizes are required for randomized clinical studies.

## Conclusion

Three months after fixed restoration, the occlusal force distribution in the molar region of the patients changed with time, which is mainly reflected in the increasing occlusal force. At the same time, occlusion time, disclusion time, and asymmetry occlusal force index tended to decrease, indicating that the distribution of occlusal force on both sides of dental arch is more ideal. Quantitative analysis of T-SCAN III can provide clinicians with more objective and precise data, allowing them to enhance the accuracy of occlusal analysis and increase the success rate of restorative treatments.

### Electronic supplementary material

Below is the link to the electronic supplementary material.

Supplementary Material 1

## Data Availability

All data generated or analysed during this study are included in this article. Further enquiries can be directed to the corresponding author.

## Abbreviations

T-SCAN III: Tekscan III
TMD: Temporomandibular disorder
TMJ: Temporomandibular joint
AOF: Asymmetry index of occlusal force
MIP: Maximum intercuspation position
OT: Occlusal time
DT: Disocclusal time
ICP: Intercuspal position
MIP: Maximal intercuspal position

## References

[1] Foz AM, Artese HPC, Horliana ACRT, Pannuti CM, Romito GA. Occlusal adjustment associated with periodontal therapy—A systematic review. J Dent. 2012;40(12). 10.1016/j.jdent.2012.09.002 22982113

[2] Santos MA, Tavares CS, Lima PT et al. Association between tooth loss and degree of Temporomandibular disorders: a comparative study. J Contemp Dent Pract. 2016;17(3). 10.5005/jp-journals-10024-1833 27207204

[3] Wang MQ, Xue F, He JJ, Chen JH, Chen CS, Raustia A. Missing posterior teeth and risk of Temporomandibular disorders. J Dent Res. 2009;88(10). 10.1177/0022034509344387 19783804

[4] Nadjmi N, Mollemans W, Daelemans A, Hemelen GV, Schutyser F, Bergé S. Virtual occlusion in planning orthognathic surgical procedures. Int J Oral Maxillofacial Surg. 2010;39(5). 10.1016/j.ijom.2010.02.002 20226628

[5] Yeliz A, Merve BG, Seçil KN, Betül KB, Cemal A. Comparison of Maximum Intercuspal contacts of Articulated casts and virtual casts requiring posterior fixed partial dentures. J Prosthodontics: Official J Am Coll Prosthodontists. 2017;26(7). 10.1111/jopr.12439 26848940

[6] Ashish Kaushik. Sumit Gahletia, Ramesh Kumar Garg, etc. Advanced 3D body scanning techniques and its clinical applications. 2022 International Conference on Computational Modelling, Simulation and Optimization (ICCMSO). 2022:352–358.

[7] Kaushik A, Garg RK. Searching the optimal parameters of a 3d scanner in surface reconstruction of a dental model using central composite design coupled with metaheuristic algorithms[J]. International Journal on Interactive Design and Manufacturing (IJIDeM); 2023.

[8] Kaushik A. Ramesh Kumar Garg. Tapping the potential of rapid prototyping techniques in creating a paradigm shift in the fabrication of occlusal splints. Rapid Prototyp J. 2023;2176–92.

[9] B KR. Computerized occlusal analysis technology and Cerec case finishing. Int J Comput Dent. 2008;11(1). 18780561

[10] Mark BKR, Mike L, John H. R. A force reproduction analysis of two recording sensors of a computerized occlusal analysis system. Cranio: J Craniomandib Pract. 2006;24(1). 10.1179/crn.2006.004 16541841

[11] C MCRF. Z, P N, P S. Validity and reliability of the T-Scan(®) III for measuring force under laboratory conditions. Journal of oral rehabilitation. 2015;42(7). 10.1111/joor.12284 25727489

[12] Ting Z, Jirapa W, Maytha S, Charukrit L, Borvornwut B. Digital occlusal analysis of pre and post single posterior implant restoration delivery: A pilot study. PloS one. 2021;16(7). 10.1371/journal.pone.0252191 PMC8253389 34214089

[13] K Y, K H. H SM, S K, Y Y. The relationship between frontal facial morphology and occlusal force in orthodontic patients with temporomandibular disorder. J Rehabil. 2000;27(5). 10.1046/j.1365-2842.2000.00531.x 10887915

[14] Yongmei H, Jie C. A study on the occlusal function of Class II division 1 malocclusion. J Practical Stomatology. 2007(03):411–3.

[15] Hitesh DRS, Geoff K. G, L CM. Midfacial and Dental Changes Associated with nasal positive Airway pressure in children with obstructive sleep apnea and Craniofacial conditions. J Clin Sleep Medicine: JCSM : Official Publication Am Acad Sleep Med 2016;12(4). 10.5664/jcsm.5668 PMC4795272 26715402

[16] d’Incau E, Couture C, Maureille B. Human tooth wear in the past and the present: tribological mechanisms, scoring systems, dental and skeletal compensations. Arch Oral Biol. 2012;57(3). 10.1016/j.archoralbio.2011.08.021 21920497

[17] Ming Z, Hui C, Xiang-rong C et al. Study on the Maximal Occlusal Force of Abutments before and after restored with rigid fixed bridge. J Oral Sci Res. 2006(06):678–80.

[18] Reddy C, Suresh S, Rama RAV. A study of change in occlusal contacts and force dynamics after fixed prosthetic treatment and after equilibration - using Tekscan III. J Indian Prosthodontic Soc. 2019;19(1). 10.4103/jips.jips_238_18 PMC6340079 30745749

[19] Qadeer S, Kerstein R, Kim RJY, Huh J-B, Shin S-W (2012). Relationship between articulation paper mark size and percentage of force measured with computerized occlusal analysis. J Adv Prosthodont.

[20] Wu B, Pu P, Zhao S (2020). Frequency-related viscoelastic properties of the human incisor periodontal ligament under dynamic compressive loading. PLoS ONE.

[21] Bhatnagar VM, Karani JT, Khanna A, Badwaik P, Pai A (2015). Osseoperception: an implant mediated sensory motor control-a review. J Clin Diagn Research: JCDR.

[22] González-Gil D, Dib-Zaitum I, Flores-Fraile J, López-Marcos J (2022). Importance of osseoperception and tactile sensibility during masticatory function in different prosthetic rehabilitations: a review. Medicina.

[23] Lloyd-Williams F, Dowrick C, Hillon D, Humphris G, Moulding G, Ireland R (2001). A preliminary communication on whether general dental practitioners have a role in identifying dental patients with mental health problems. Br Dent J.

[24] Bozhkova TP (2016). The T-SCAN system in evaluating occlusal contacts. Folia Medica.

[25] de Prado I, Iturrate M, Minguez R, Solaberrieta E. Evaluation of the accuracy of a system to align occlusal dynamic data on 3D digital casts. BioMed Research International. 2018;2018. 10.1155/2018/8079089 PMC6011109 29977917

[26] Wang C, Yin X (2012). Occlusal risk factors associated with temporomandibular disorders in young adults with normal occlusions. Oral Surg oral Med oral Pathol oral Radiol.

[27] Baldini A, Nota A, Cozza P (2015). The association between occlusion time and temporomandibular disorders. J Electromyogr Kinesiol.

[28] Cheng H-J, Geng Y, Zhang F-Q. The evaluation of intercuspal occlusion of healthy people with T-Scan II system. Shanghai J Stomatology. 2012;21(1). 22431060

[29] Thumati P, Manwani R, Mahantshetty M (2014). The effect of reduced disclusion time in the treatment of myofascial pain dysfunction syndrome using immediate complete anterior guidance development protocol monitored by digital analysis of occlusion. Cranio®.

