# Supplementary material for: Use of T-Scan III in analyzing occlusal changes in molar fixed denture placement
Source: BMC Oral Health. 2024 Feb 22;24:264. doi: 10.1186/s12903-024-04014-1 (PMC10885451; doi:10.1186/s12903-024-04014-1)
Supplement: Supplementary file 1 — Supplementary Material 1 [file 12903_2024_4014_MOESM1_ESM.doc]

Supplementary file: Questionnaire 1: The patients' responses to the standard questionnaire used in the study

Q1 Do you feel comfortable when you bite on your teeth lightly?

| 1 | 2 | 3 | 4 | 5 |
| --- | --- | --- | --- | --- |

Very uncomfortable I feel comfortable

Q2 Do you feel comfortable when you bite down hard on your back teeth?

| 1 | 2 | 3 | 4 | 5 |
| --- | --- | --- | --- | --- |

Very uncomfortable I feel comfortable

Q3 Do your back teeth contact each other evenly when you bite down hard?

| 1 | 2 |
| --- | --- |

No even contact Yes, even contact

Q4 Do you feel any pain when you bite down hard on your back restorative teeth?

| 1 | 2 | 3 | 4 | 5 |
| --- | --- | --- | --- | --- |

Very uncomfortable I feel comfortable

Q5 Do your back teeth contact each other evenly when you bite down hard in restoration region?

| 1 | 2 |
| --- | --- |

No even contact Yes, even contact

Q6 Do you feel pain/tenderness when you bite hard?

| 1 | 2 | 3 | 4 | 5 |
| --- | --- | --- | --- | --- |

Very painful No pain

Q7 Do you feel any pain when you bite down hard on your back teeth in restored/missed tooth region?

| 1 | 2 | 3 | 4 | 5 |
| --- | --- | --- | --- | --- |

Very painful No pain

Q8 Do you feel pain at the temporomandibular joint while chewing in the restored/missed tooth region?

| 1 | 2 | 3 | 4 | 5 |
| --- | --- | --- | --- | --- |

Very painful No pain

Q9 How well were you able to chew though food like meat?

| 1 | 2 | 3 | 4 | 5 |
| --- | --- | --- | --- | --- |

Cant chew well Chew very well
